# Within Patient Radiological Comparative Analysis of the Performance of Two Bone Graft Extenders Utilized in Posterolateral Lumbar Fusion: A Retrospective Case Series

**DOI:** 10.3389/fsurg.2015.00069

**Published:** 2016-01-25

**Authors:** Geoffrey Stewart, Gary B. Gage, Gary Neidert, Huston Davis Adkisson

**Affiliations:** ^1^The Spine and Scoliosis Center, Orlando, FL, USA; ^2^ISTO Technologies, Inc., St. Louis, MO, USA; ^3^Innovative Analytics, Inc., Kalamazoo, MI, USA

**Keywords:** bone graft extender, hyaluronic acid, spine fusion, bone marrow aspirate, endochondral ossification, biomaterial scaffold

## Abstract

Two bone graft extenders differing in chemical composition were implanted contralaterally in 27 consecutive patients undergoing instrumented posterolateral lumbar fusion as standard-of-care. Bone marrow aspirate and autogenous bone graft were equally combined either with β-tricalcium phosphate (β-TCP) or a hybrid biomaterial [containing hyaluronic acid (HyA) but lacking a calcium salt] and implanted between the transverse processes. Fusion status on each side of the vertebrae was retrospectively graded (1–5 scale) on AP planar X-ray at multiple visits as available, through approximately 12 months. Additionally, consolidation or resorption since prior visit for each treatment was recorded. Sides receiving β-TCP extender showed marked resorption prior to bone consolidation during the first 6 months. By contrast, sides receiving the hybrid biomaterial containing integrated HyA showed rapid bone consolidation by week 6–8, with maintenance of initial bone volume through 12 months. Fusion grade was superior for the hybrid biomaterial, differing significantly from β-TCP at day 109 and beyond. Fusion success at >12 months was 92.9 vs. 67.9% for the hybrid biomaterial and β-TCP-treated sides, respectively. The hybrid biomaterial extender demonstrated a shortened time-to-fusion compared to the calcium-based graft. Mode of action has been demonstrated in the literature to differ between these compositions. Therefore, choice of synthetic biomaterial composition may significantly influence the mode of action of cellular events regulating appositional bone growth.

## Introduction

Posterolateral spine fusion with pedicle screw stabilization is a common surgical procedure for treatment of patients with intractable back pain caused by intervertebral disc degeneration with associated spondylolisthesis and/or stenosis. Fusion of the involved spinal segments is achieved by bone generation on either side of the vertebral column between the affected transverse processes (gutters) facilitated by placement of bone grafts after decortication of the transverse processes. It is fundamentally accepted that in the absence of added cancellous bone graft (containing viable osteoblastic and endothelial elements), the local microenvironment of the posterolateral gutters is challenged in its capacity to support large volumes of newly formed bone (1, 2).

Historically, autogenous marrow-rich iliac crest bone graft has been the “gold standard” of treatment used to promote bone growth in posterolateral fusion because of its proven osteoconductive, osteogenic, and osteoinductive properties (3). However, complication rates for this procedure remain high, with donor site morbidity related to nerve injury, excessive blood loss, and deep wound infection being most commonly reported (4, 5). Of more practical importance is the limited volume of iliac crest available for multi-level fusion procedures and for individuals in whom previous surgeries have been completed. In an attempt to either reduce or eliminate dependence on the use of autogenous iliac crest bone graft, a variety of synthetic osteoconductive extenders have been developed and widely investigated (6, 7). While multiple level II studies have demonstrated equivocal fusion and clinical outcomes for use of rhBMP-2 with Mastergraft in single-level instrumented posterolateral lumbar fusion as a substitute for iliac crest bone graft (8–11), recent scrutinization of product safety and off-label use questions whether the benefits of rhBMP-2 outweigh its cost and associated patient risks (12, 13).

Calcium sulfate and calcium phosphate ceramic bone substitute materials, including hydroxyapatite and β-tricalcium phosphate (β-TCP) or their composites, as well as the bioglasses, are widely utilized to minimize or replace the harvest of patient-derived bone (7, 14–19). While these ceramic-based materials were designed to mimic the inorganic phase of native bone, inherent disadvantages in their physical properties, variability in biochemical characteristics, resorption and bone consolidation rates can increase the risk for pseudarthrosis and implant failure (19–25). Further, recent *in vitro* studies have demonstrated an inefficient binding to and retention of human osteoprogenitor cells loaded onto a variety of commercially available ceramic-based products differing in their calcium phosphate chemistries (26, 27). It, therefore, remains unknown whether these differences may ultimately affect the efficacy of ceramic-based bone grafts used to extend autogenous bone utilized in posterolateral fusion procedures (26).

A hybrid composite of resorbable polymer and natural polysaccharide offers predictable, complete replacement by new bone, while exhibiting unique biological and physical characteristics intended to partner with the body’s own healing potential to foster bony ingrowth. This novel biomaterial, known as InQu^®^ Bone Graft Extender & Substitute, has proved clinically effective in posterior interbody lumbar fusion procedures (28), in addition to the successful treatment of long bone defects (Harris and Adkisson, in preparation) and the reconstruction of high-risk foot and ankle deformities secondary to diabetes (Charcot reconstruction; Kerzner, in preparation). The hybrid biomaterial provides a compressive resistant, porous osteoconductive scaffold made of poly (d, l-lactide-*co*-glycolide), within which hyaluronic acid (HyA) is entangled. To date, no clinical study has explored the safety and effectiveness of this radiolucent extender to promote lumbar posterolateral arthrodesis.

The purpose of the present retrospective clinical case series was to compare fusion grade and consolidation/resorption characteristics of two commercially cleared synthetic bone graft substitute devices utilized within the same patients, per physician discretion, on opposite sides of the fusion construct as autograft extenders for instrumented posterolateral lumbar fusion. Additionally, a time-to-fusion analysis between these two products was evaluated. It was hypothesized that differences in biomaterial composition may reveal differences in the rate at which new bone was formed.

## Materials and Methods

### Study Design

This is a single center, non-randomized, case series comprising 27 consecutive patients having undergone single- and/or multi-level posterolateral lumbar fusions with segmental spinal instrumentation utilized bilaterally. A retrospective data collection effort to assess radiographic outcomes conducted under IRB oversight (waiver of consent) was requested of the Western Institution Review Board (WIRB) and approved. No additional clinical outcomes were assessed as part of this study.

Surgeries were performed between September 2008 and May 2009 in which InQu^®^ (ISTO Technologies, St. Louis, MO, USA) and β-TCP (TheriGraft^®^ TCP Putty Bone Void Filler, Therics, LLC, Princeton, NJ, USA) were applied contralaterally as autograft extenders per physician discretion as described below. This was a standard-of-care approach to spine fusion utilizing two devices with 510k clearances in association with pedicle screw stabilization. Radiographic follow-up standard-of-care included anterior/posterior X-rays at various intervals post-surgery to 15 months. Because computed tomography (CT) was not medically necessary for any of the study subjects, no CT exams were ordered as part of clinical radiographic follow-up.

### Patient Demographics

The study population encompassed 27 subjects (18 males; 9 females) averaging 62.7 years of age (range, 26–86 years; median 67 years). No subjects were excluded from analysis. All comorbidities were recorded, including fusion risk factors and patient diagnoses prior to surgery. One subject received treatment at two different contiguous fusion levels of the vertebrae; thus, data for 28 contiguous fusion levels and 56 distinct fusion segments were captured.

### Surgery

Local bone removed from the laminae and spinous processes in the region of spinal canal stenosis was morcelized and split equally between treatment sides after meticulous removal of covering soft tissues. Approximately 10 cc of posterior iliac crest was then harvested and similarly morcelized and divided among treatment sides. Next, 10 cc of graft extender (either InQu or TCP) was mixed with 6 cc of bone aspirate harvested from the posterior iliac crest and immediately applied to morcelized bone (obtained from the iliac crest and locally). Once stabilization of the target level(s) was completed using transpedicular screw/rod instrumentation with cross-links, decortication of the transverse processes was performed. Equivalent amounts of the final extender construct were placed on either side of the posterolateral gutters – β-TCP on the left and InQu hybrid biomaterial on the right. All surgical procedures were performed per the surgeon’s routine standard care. If spondylolisthesis was present, no forceful reduction was attempted. If circumferential fusion was indicated (*n* = 16), only iliac crest bone graft was used within the interbody fusion device. No additional graft material was placed anterior to the cage. Patients were seen for routine follow-up assessment and anterior/posterior (A/P) and lateral radiographs as per the standard-of-care, usually at approximate intervals of 2 weeks after surgery (baseline), 3, 6, and 12 months. These post-operative radiographs were collected between 9/18/2009 and 7/22/2010.

### Outcomes

**Figure 1:**
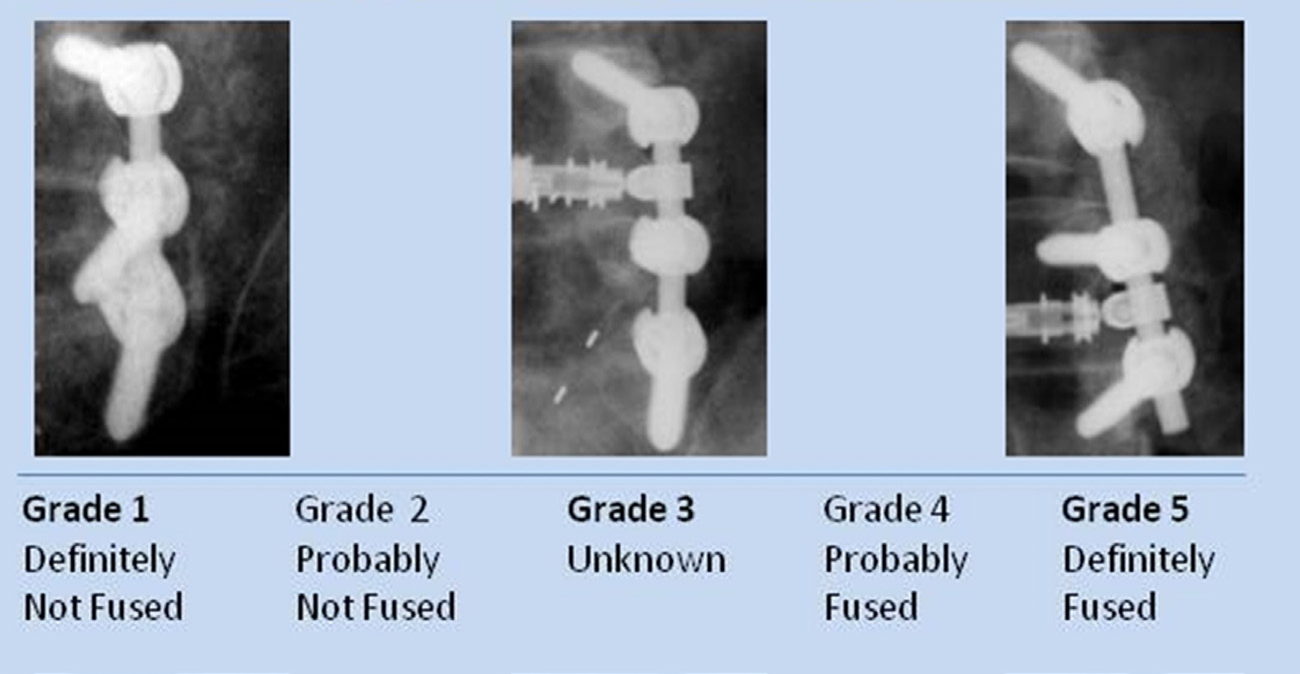
**Examples of fusion grade definition**.

The physician read each of the radiographs (unblinded) and recorded his fusion assessment using a fusion grade scale. Each fusion side (the whole contiguous fusion segment whether multi-level or single-level fusion) was evaluated separately for fusion grade (5 = Definitely fused; 4 = Probably fused; 3 = Indeterminate; 2 = Probably not fused; 1 = Definitely not fused) (Figure 1). Sequential radiographs were graded for graft consolidation/resorption as compared with the prior visit. Demographic and baseline data were tabulated and summarized for the whole population.

### Statistics

The first primary outcome measure determined the difference in fusion grades between treatment type over time using a mixed model, repeated measures, covariate analysis in SAS (SAS Institute, Inc., Cary, NC, USA). Once the final model was determined, a predicted regression line over time was developed for each treatment. Predicted mean fusion grade difference between treatments over time was compared statistically and represented graphically.

The second assessment tabulated fusion for each treatment type at various post-operative time intervals. This was performed by comparing percentages of fusions categorized as “probably/definitely” fused, “indeterminate,” and “probably/definitely” not fused at defined time intervals: days 21–90; 91–180; 181–360; and >360. A “last observation carried forward” procedure was used to impute missing fusion rates. Statistical comparisons between treatments were performed using the Friedman Chi-square Test at each time interval and for the overall interval.

The third primary assessment was a Kaplan–Meier time-to-fusion analysis conducted for both treatments. Time to first “Probably/Definitely” fused (fusion grade of 4 or 5) was computed for each fusion segment using the Kaplan–Meier estimate for each treatment. The median time-to-fusion was estimated across time (days to fusion) for each bone graft extender. The treatment curves were compared. Fusion segments (grades 1–3) that never completely fused >360 days were considered treatment failures.

A secondary outcome measure was radiologic consolidation/resorption of the fusion construct. Consolidation since prior visit was graded as an increase (+1 increment); resorption since prior visit as a decrease (−1 decrement); and no change since prior visit was graded as zero. These values were added sequentially for plotting purposes. The value at each assessment was added to the prior assessment, and the values were plotted on a graph separately for each fusion segment for both treatments. The trends were provided graphically descriptively without statistical analyses.

## Results

### Demographics and Baseline

Twenty-seven subjects comprising 28 contiguous treatment levels (bilateral segments), and thus 56 distinct individual fusion segments, were identified and their data were collected retrospectively in case report forms by site personnel. This cohort represented a case series of consecutive patients requiring lumbar arthrodesis, and therefore included a heterogeneous mix of patients with respect to general health status, number of levels fused and concurrent use of interbody spacers. Fusion levels within each bilateral segment ranged from 1 to 6 levels. One subject had two bilateral segments treated that were non-contiguous (L2-3 and L5-S1). Each treated segment was evaluated separately. Therefore, a total of 28 bilateral fusion segments or 56 individual fusion segments were evaluated. All fusions utilized pedicle screws and rods with cross-links. Sixteen subjects (59.2%) underwent concurrent posterior interbody fusion at one or more levels with posterolateral fusion in this study (Table 1).

**Table 1:** **Posterolateral (PL) fusion levels and concurrent interbody (IB) fusions**.

Number of patients	Number of PL fusion levels	Number of concurrent IB levels
1	1	0
7	2	0
1	3	0
2	4	0
5	1	1
4	2	2
5	2	1
1	4	1
1	6	1

The demographics and baseline characteristics are identical for both treatments (Table 2). Mean age of the population was 62.7 years (range 26–86, SD, 13.44 years). The subjects were primarily male (70.4%), non-smokers (92.6%), and recipients of two-level fusions (57.1%). The population was generally overweight by US standards with a mean BMI of 29.52 (range 22.3–41, median 27.8), but one-third of the population was considered obese.

**Table 2:** **Baseline patient demographics**.

Baseline variable	Number subjects (%)
	*N* = 27
**GENDER**	
Male	19 (70.4%)
Female	8 (29.6%)
**BMI**	
≤30	18 (66.7%)
>30	9 (33.3%)
**AGE**	
≤60 years	8 (29.6%)
>60 ≤ 70 years	13 (48.1%)
>70 years	6 (22.2%)
**SMOKER**	
Yes	2 (7.4%)
No	25 (92.6%)
	**Fusion segments**
	***N* = 28^a^**

**NUMBER OF FUSION LEVELS**	
Single	7 (25.0%)
Double	16 (57.1%)
3+ levels	5 (17.9%)

Body mass index (BMI) of 18.5–24.9 is considered normal weight; BMI of 25–29.9 is considered overweight; BMI of 30 and above is considered obese. http://en.wikipedia.org/wiki/Body_mass_index

*^a^One subject had two fusion levels that were not contiguous; each fusion segment in this subject was evaluated separately*.

Baseline co-morbid chronic diseases potentially affecting arthrodesis were collected and tabulated (Table 3). The population was predominantly free of risk factors affecting bone healing: 66.7% were without diabetes; 92.6% were without chronic obstructive pulmonary disease (COPD); and 70.4% were without coronary artery disease. No patients reported chronic renal disease or steroid use. Non-steroidal anti-inflammatory drugs (NSAIDs) were discouraged prior to surgery until 6 months after surgery. Only two patients were recorded as chronic tobacco users and nine were recorded as receiving treatment for diabetes.

**Table 3:** **Baseline co-morbid chronic diseases**.

Comorbidities	Number subjects (%)
	*N* = 27
**DIABETES**	
Yes	9 (33.3%)
No	18 (66.7%)
**CORONARY ARTERY DISEASE (CAD)**	
Yes	8 (29.6%)
No	19 (70.4%)
**CHRONIC OBSTRUCTIVE PULMONARY DISEASE (COPD)**	
Yes	2 (7.4%)
No	25 (92.6%)
**CHRONIC RENAL FAILURE**	
Yes	0 (0.0%)
No	27 (100.0%)
**STEROIDS USE**	
Yes	0 (0.0%)
No	27 (100.0%)
**NSAIDs USE**	
Yes	0 (0.0%)
No	27 (100.0%)

*NSAIDs, non-steroidal anti-inflammatory drug*.

Concomitant spinal diagnoses are presented in Table 4. Patients in this study were undergoing decompressive laminectomy and posterolateral lumbar fusion for spinal stenosis (88.9%), herniated nucleus pulposus (22.2%), or both. Concomitant spinal diagnoses included spondylolisthesis (37%); degenerative disc disease (22.2%); prior laminectomy (11.1%); pseudarthrosis (3.7%); and scoliosis (3.7%).

**Table 4:** **Concomitant spinal diagnoses**.

Concomitant spine diagnoses	Number patients (%)
	*N* = 27
**SPINAL STENOSIS**	
Yes	24 (88.9%)
No	3 (11.1%)
**SPONDYLOLISTHESIS**	
Yes	10 (37.0%)
No	17 (63.0%)
**HERNIATED NUCLEUS PULPOSUS**	
Yes	6 (22.2%)
No	21 (77.8%)
**DISC DEGENERATION**	
Yes	6 (22.2%)
No	21 (77.8%)
**SCOLIOSIS**	
Yes	1 (3.7%)
No	26 (96.3%)
**POST-LAMINECTOMY**	
Yes	3 (11.1%)
No	24 (88.9%)
**PSEUDOARTHROSIS**	
Yes	1 (3.7%)
No	26 (96.3%)

### Analysis of Predicted Mean Fusion Grade Differences Over Time

**Figure 2:**
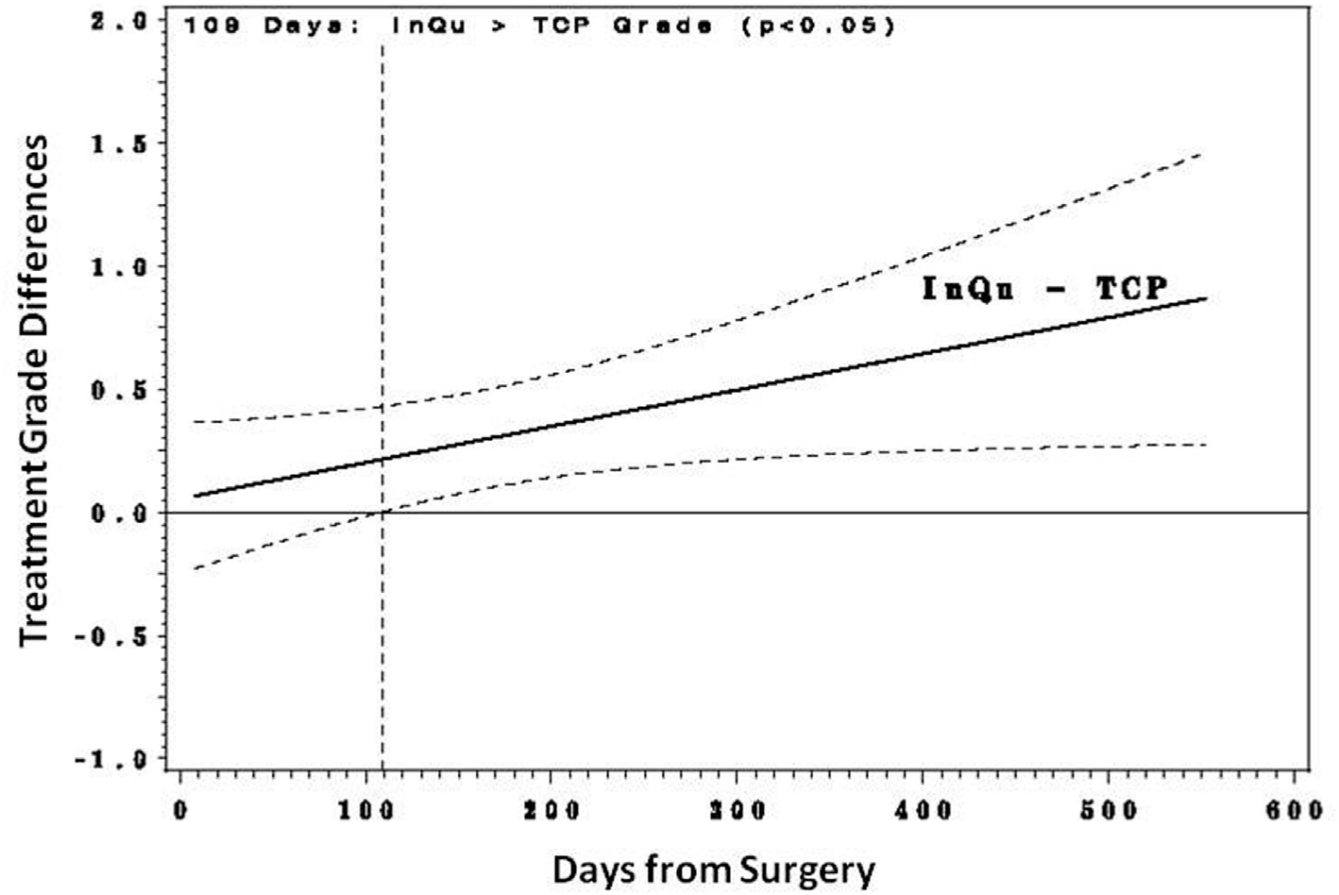
**Predicted mean differences with 95% confidence intervals**. From day 109 forward, the predicted mean fusion grade (grade 4 probably fused/grade 5 definitely fused) for segment receiving InQu extender was found to be statistically significantly (*p* < 0.05) better than the predicted mean for those sides receiving β-TCP extender.

**Figure 3:**
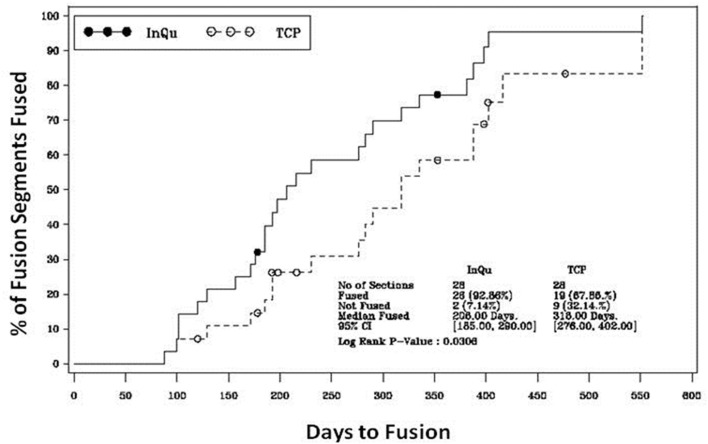
**Kaplan–Meier analysis of time-to-fusion**. The log-rank test of the homogeneity of the Kaplan–Meier curves showed that the time-to-fusion for the InQu-treated side at 206 days was significantly (*p* < 0.0309) shorter than the β-TCP curve at 318 days.

Fusion was assessed for each (left and right) posterolateral contiguous fusion segment according to the graft extender used (*n* = 28 InQu treated; *n* = 28 β-TCP treated). Fusion grade scores were examined with a repeated measures regression analysis with several covariates, including number of levels treated, days from surgery, subject age, gender, BMI, and comorbidities to fusion (tobacco use, diabetes, COPD, spondylolisthesis, herniated disc, DDD). Only “treatment by days from surgery” was a significant factor. This interaction was explored (Figures 2 and 3) by comparing the treatment’s predicted mean fusion grade responses at selected time points that spanned the days from surgery. Although during the initial healing phase (<day 109) the predicted mean fusion grades for the InQu-treated side were greater than that for the β-TCP-treated side, statistically significant differences were not experienced. However, from day 109 forward, the predicted mean fusion grade for InQu was found to be statistically significantly (*p* < 0.05) greater than the predicted mean for β-TCP.

### Fusion Assessment at Various Time Intervals

Fusion rates were assessed at four time periods (Table 5). A last observation carried forward procedure was used to impute missing rates. Due to fragmentation of the small sample size across five categories that dilute the analysis, “Definitely Fused”/“Probably Fused” were collapsed into a single category, “Fused”; similarly, “Definitely Not Fused”/“Probably Not Fused” were collapsed into – “Not Fused.” When comparing the fusion outcomes across all time intervals using the Friedman (Chi-Square) test, statistically significant overall differences (*p* < 0.0031) were observed. A higher percentage of InQu-treated fusion segments demonstrated successful fusion results than β-TCP-treated fusion segments. By days 181–360, the significant difference (*p* < 0.05) was 75% fused (InQu) vs. 50% fused (β-TCP); and for days >361, the significant difference was 92.9% fused (InQu) vs. 67.9% fused (β-TCP) (*p* < 0.02).

**Table 5:** **Fusion assessment outcomes by time interval**.

Treatment	Assessment	Days 21–90	Days 91–180	Days 181–360	Days >361
InQu, *n* = 28	Not fused	26 (92.6%)	8 (28.6%)	2 (7.1%)	0 (0.0%)
	Indeterminate	1 (3.6%)	11 (39.3%)	5 (17.9%)	2 (7.1%)
	Fused	1 (3.6%)	9 (32.1%)	21 (75.0%)	26 (92.9%)
TCP, *n* = 28	Not fused	25 (89.3%)	11 (39.3%)	6 (21.4%)	3 (10.7%)
	Indeterminate	2 (7.1%)	13 (46.4%)	8 (28.6%)	6 (21.4%)
	Fused	1 (3.6%)	4 (14.3%)	14 (50.0%)	19 (67.9%)

Generalized overall Friedman (Chi-Square) Test		Value, *p*-value	Value, *p*-value	Value, *p*-value	Value, *p*-value
		0.10, 0.7555	2.00, 0.1568	3.96, 0.0465	5.95, 0.0147

		Value, *p*-value			
		8.75, 0.0031			

Two subjects were not fused on both the β-TCP-treated and the InQu-treated segments. Zero segments were not fused on the InQu-treated side only, whereas seven segments were not fused on the β-TCP-treated side only. Of the two subjects that did not fuse on either side, one was treated from T12-S1 and the fusion was graded as 3 – Indeterminate – at the last visit recorded, almost 1-year post-operative. In the same subject at that visit, the InQu-treated side showed increasing consolidation, but the β-TCP side showed increasing resorption since prior visit. The second subject with fusion failure on both sides was graded a 3 – Indeterminate – on both sides, with increasing consolidation observed for both treatments at 5 months, the last recorded visit.

Of the 11 subjects identified as either active smokers or having diabetes, no fusion failures were noted for either treatment. However, in four fusion segments (three patients) receiving β-TCP extender, an “Indeterminate” fusion (Grade 3) was recorded. The opposite side receiving InQu extender in these patients presented radiographically as “Probably Fused” (Grade 4) in four segments, with the last being “Indeterminate” (Grade 3) and was not considered statistically significantly different.

### Time-to-Fusion Analysis

The results of the Kaplan–Meier analysis of Time-to-Fusion are displayed in Table 6 and illustrated in Figure 3. Using the collapsed categories of Fused, Indeterminate, and Not fused, a “worst-case” scenario counted all “Indeterminate” assessments as fusion failures. Comparison of the fusion assessment showed that the InQu-treated segments fused significantly faster than the β-TCP-treated sides. The estimated median Time-to-Fusion for InQu and β-TCP were 206 and 318 days, respectively (*p* < 0.0309).

**Table 6:** **Time-to-fusion**.

Characteristic	Statistics	InQu	TCP
Time-to-first probably/definitely fused (days)	*N*	28	28
	*n* (fusion)	26	19
	*N* (no fusion)	2	9
	Median days to first fusion^a^	206	318
	25th percentile^a^	14	192
	75th percentile^a^	355	402

	Log-rank test	4.6561	
	*p*-value^b^	0.0309	

*^a^The median, 25th percentile, and 75th percentile are estimated from Kaplan–Meier survival curve*.

*^b^*p*-value calculated based on Chi-Square (1 degree of freedom)*.

### Graft Resorption/Consolidation Assessment

**Figure 4:**
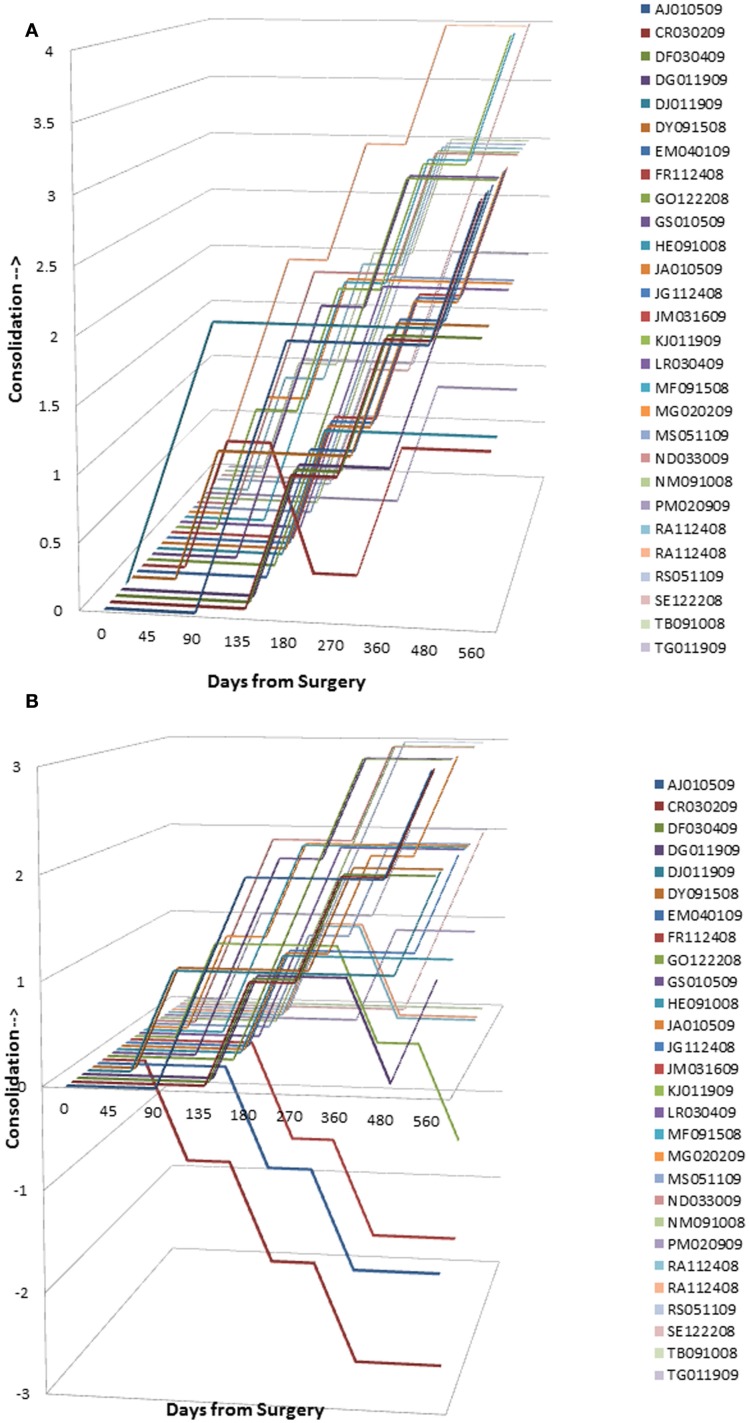
**(A)** InQu resorption/consolidation assessment by subject over time. Each line lies in the upper half of the graph demonstrating that there is no significant initial resorptive phase, only intervals of increasing consolidation over time. **(B)** β-TCP resorption/consolidation assessment by subject over time. A flat line demonstrates for most of the β-TCP-treated fusion segments between days 91–135 no change in radiographic appearance from surgery. Once the mineral content has been cleared, an increase in the height of the plotted lines (consolidation) was observed for one or more time intervals in 21 of 28 fusion segments. However, from day 135 and on, 7 of 28 fusion segments (25%) showed a steady decline in line height toward the negative portion of the graph, indicating that more resorption had occurred than consolidation.

Graphical analysis of graft resorption/consolidation (as described previously) was plotted as the format of the data did not lend itself to statistical analysis. The plots show graphically the differences between treatment sides. For sides receiving InQu extender (Figure 4A), all the lines lie in the upper half of the graph, demonstrating that there is no significant initial resorptive phase, only intervals of increasing consolidation over time. Prior to day 90, most of the lines are flat, demonstrating that there is no radiographically observable difference from surgery. Not only is InQu radiolucent, but also cartilage growth – an early step in InQu’s mechanism of action “endochondral ossification” – is not easily observed radiographically on plain films. However, bony consolidation is observable on plain radiographs concurrently with cartilage calcification and formation of woven bone. Between days 91 and 135, all the InQu fusion segment lines showed a steady increase, and progressive consolidation was generally observed to 360 days and beyond.

**Figure 5:**
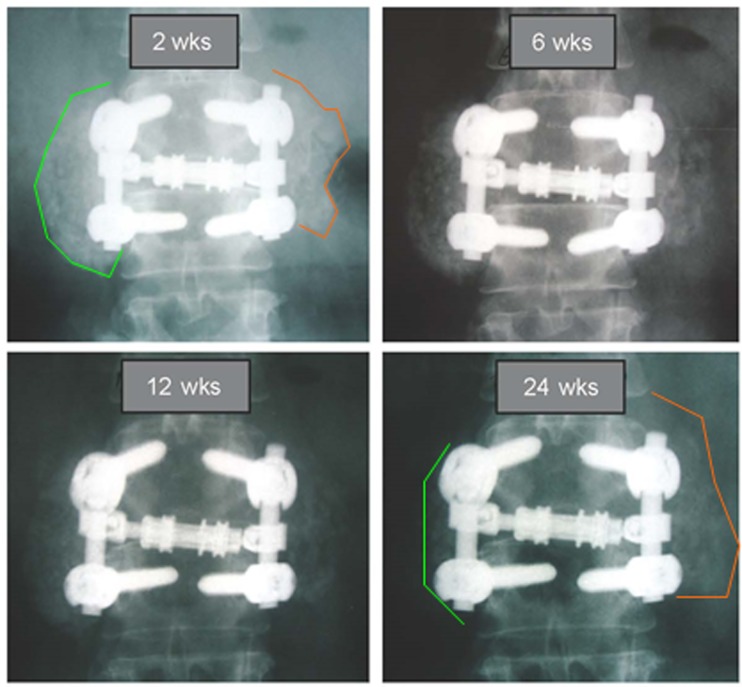
**Comparative radiography depicting resorption and consolidation of two bone graft extenders**. A 46-year-old female with history of active tobacco use and diabetes underwent PL fusion of the lumbar spine at L2-3 and L5-S1. AP radiographs at L2-3 are shown over time. Left side received bone autograft, BMA, and β-TCP putty. Right side received equal mix of bone autograft, BMA, and InQu putty. Sequential assessment over 24 weeks illustrates marked resorption of ceramic bone graft, whereas the opposite side receiving hybrid extender showed consolidation of applied graft at 6 weeks, with sustained bone volume at 24 weeks.

For sides receiving β-TCP extender (Figure 4B), a different picture was observed that is consistent with the β-TCP mechanism of action. A flat line demonstrates for most of the β-TCP fusion segments between days 91 and 135, no change in radiographic appearance from surgery. Given the high radiographic signal intensity of β-TCP, it is difficult to estimate graft consolidation with surrounding tissue until the mineral content has been cleared by osteoclastic activity and replaced by new bone. Once complete, an increase in the height of the plotted lines (consolidation) was observed for one or more time intervals in 21 of 28 fusion segments. However, from day 135 and forward, seven of 28 fusion segments (25%) showed a steady decline in line height toward the negative portion of the graph, indicating that more resorption had occurred than consolidation. A representative example of this resorption phase, identified only for sides receiving β-TCP extender, is illustrated in Figure 5. In this subject (46 year-old female with diabetes) sequential radiographs document marked resorption of implanted β-TCP between weeks 2 and 24, whereas the contralateral side receiving hybrid extender demonstrated radiographic evidence of consolidation as early as week 6 post-treatment.

There were only a limited number of actual data points at days >360; the rest of the data at this time point were imputed in a last-observation-carried-forward (LOCF) analysis. It is unclear if an actual plateau had been attained for each patient in either treatment group or whether one or both groups would continue to consolidate further.

### Safety

Two adverse events were identified in this patient sample upon radiographic review of day 270–360 radiographs. One patient had radiographic evidence of a fractured screw, although fusion appeared solid. The second patient developed a large synovial cyst at an adjacent level with neurocompression; the patient was subsequently treated with surgical decompression and instrumented fusion. In the latter case, surgical exploration of the fusion segment confirmed solid fusion. No serious adverse events relating specifically to the use of β-TCP or InQu were reported during the study.

## Discussion

Bone graft substitutes are commonly used to extend the volume of available autogenous bone harvested in major reconstructive spine procedures (7, 15). While ceramic-based osteoconductive scaffolds account for a majority of the synthetic bone substitute materials, it is unclear whether inorganic mineral is essential for – or contributes favorably to – bone formation. Preclinical studies have shown that persistence of hydroxyapatite containing grafts may interfere with progressive bone formation (20, 21). Similarly, too rapid resorption of calcium sulfate (20, 24) and fragmentation of partially resorbed β-TCP may be associated with failed arthrodesis both in animals and man (20, 29). Further, Hsu et al. (26) and Murphy et al. (27) independently demonstrated unfavorable biocompatibility characteristics for a variety of ceramic-based bone graft substitutes with respect to cell binding, retention, and proliferation. It was concluded that only osteoconductive scaffolds, which allow for high rates of preosteoblast adhesion, cell survival, and proliferation may be expected to yield the most predictable, effective clinical performance.

The present study retrospectively compared overall fusion success of a β-TCP ceramic bone graft and a novel non-ceramic biosynthetic scaffold-containing integrated HyA. These 510k cleared devices were evaluated side-by-side in a series of patients requiring instrumented posterolateral lumbar fusion. Plain film radiographic assessment was used to grade posterolateral fusion over time. These data were assessed for predicted mean differences between treatments over time using a mixed model, repeated measures covariate analysis, which demonstrated statistically significantly higher fusion grades for InQu extender over β-TCP extender at day 109 and beyond. Additionally, Kaplan–Meier analysis of Time-to-Fusion demonstrated a substantial time advantage (112 days faster) in the assimilation and fusion process for those segments receiving InQu over β-TCP. The radiographic description of resorption/consolidation between assessment windows revealed that 25% of the β-TCP segments failed to trend toward consolidation, whereas all segments receiving the hybrid polymer demonstrated consolidation at one or more assessment windows, even in eight of nine (88.9%) subjects receiving clinical treatment for diabetes.

The mechanism of bone formation supported by the application of InQu to osseous defects has been reported previously. Histological assessment of healing for cancellous metaphyseal defects of the distal femur in rabbits presented clear evidence for endochondral bone growth via the migration and differentiation of mesenchymal stem cells from surrounding bone marrow (30). At 3 months, the porous structure of InQu was replaced by calcified cartilage and newly woven bone, which by 6 months had remodeled to form mature lamellar bone. Histological observation of sham defects in rabbits revealed an abnormal fatty appearance to the marrow space occupying these defects, with a futile attempt at revascularization. Further confirmation of this mechanism of action was recently obtained in the Boden model for posterolateral lumbar fusion using a mixture of InQu granules and ICBG (50:50 ratio) (31). Vascularization of a cartilage intermediate was accompanied histologically by calcification of the cartilage matrix, which ultimately was replaced by woven bone from invading osteoprogenitor cells originating from the vasculature (31). It is noteworthy that the formation of cartilagenous matrix does not lend itself to radiographic characterization before it has undergone terminal differentiation through bony replacement. Fusion (Grade 4/5) in the present study was generally first observable for the InQu-treated fusion segments at day 206, but not until day 318 for the β-TCP-treated fusion segments, and ultimately, the degree of fusion success at >day 360 was much higher in the InQu-treated group (92.6% InQu vs. 67.9% β-TCP, *p* < 0.02).

The absorption and bony replacement of β-TCP-based grafts involves a biologically distinct mechanism whereby osteoclasts, formed through macrophage fusion, adhere to the β-TCP surface to remove mineral in preparation of osteoblast adherence and the deposition of newly mineralized bone matrix (20, 22, 32). Histological review of the *in vivo* resorption characteristics of β-TCP implanted into osteochondral defects in rabbits suggested that osteoclastic resorption and fragmentation of the osteoconductive scaffold minimized the volume of regenerated bone (20). This observation of a loss of template bone volume is consistent with a previous report by Hollinger et al. (33), placing into question whether β-TCP-based bone grafts should be used as extenders in reconstructive spine procedures where large volumes of new bone are required to stabilize implant hardware. Radiographs from the present study captured the resorption of β-TCP extender as a reduction in radiographic intensity, which appeared as early as 6–12 weeks postoperatively. Interestingly, this resorption process progressed further in a handful of cases to reflect a significant loss of bone volume between months 6 and 12 of follow-up. Extensive graft resorption accompanied by limited production of new bony ingrowth has the disadvantage of providing poor stability to the fusion segment, which could lead to future complications requiring intervention.

Other potential disadvantages to the use of ceramic bone graft substitutes for spine fusion are their reported friability, low impact resistance (19, 22), and the radiographic challenge posed by their intrinsic radiopacity (34). By contrast, InQu is radiolucent and permits progressive visualization on plain film X-ray of the incorporation/consolidation of newly formed bone. Furthermore, it should be emphasized that InQu-treated segments appeared radiographically to either maintain or increase their initial bone volume with fusion progression. This observation suggests that InQu provides a stable porous structure that retains its bulking properties under compression in the posterolateral gutters. Unlike ceramic bone grafts, the backbone structure of InQu (PLGA) biodegrades through random hydrolytic scission of ester bonds, independently of cellular activity (33, 35, 36). Preclinical studies have demonstrated a tissue residence time of 3–6 months for InQu granules that were mixed equally with ICBG for posterolateral lumbar fusion (31).

The role of HyA in embryonic development and general wound healing is well recognized (37–39). However, few studies have explored the potential contribution of HyA to bone healing and remodeling (39–41). Sasaki and Watanabe (39) were the first to report the healing effect of HyA on bone growth in rats using a bone marrow ablation model (39). Importantly, HyA is reported to support renewal of hematopoietic stem cells by increasing cytokine production (42). In line with these results, Sasaki and Watanabe presented histological evidence for normal reconstitution of marrow cavities in half the time observed for sham controls (saline), following the administration of high-molecular weight HyA (39). CD34^+^ endothelial progenitor cells may play a critical role in revascularization following bone marrow ablation injury and treatment with HyA. This hypothesis is supported by the recent findings of Raines et al. (40), who reported neovascularization by exogenous high-molecular weight HyA following bone marrow ablation in rats. Additional studies have reported that scaffolds created from chemically modified HyA enhance mesenchymal stem cell binding and yield greater quantities of new bone in osteochondral defects created in the rabbit knee when compared to defects receiving resorbable PLGA scaffolds alone (43, 44). Solchaga et al. (43, 44) found that tissue-engineered scaffolds made from HyA supported endochondral ossification, recapitulating embryologic events recognized to mediate long bone growth and fracture repair. In light of these findings, it is speculated that the hybrid biomaterial (InQu) used in the present clinical study supports bone regeneration by creating a local environment conducive to the attachment, proliferation, and differentiation of the body’s own stem cells. Both endothelial and osteogenic progenitor cells retain specific cell surface receptors for HyA, which mediate cell survival, proliferation, and differentiation (38, 45–47), through which cell-receptor coupling may contribute to the shortened time-to-fusion borne out by the current data.

Several weaknesses of the study should be mentioned. This was a retrospective study in a small patient group in a clinical practice setting, and not a research setting. Both graft extenders were commercially available and utilized at the sole discretion of the attending physician. Data collection was performed according to physician’s standard-of-care. Therefore, neither special measures of 3-D CT reconstruction were performed nor were clinical outcomes data pertaining to pain and function collected via a validated instrument. While evaluations (radiological assessment of fusion and resorption/consolidation) were performed by the attending physician (GS) in an unblinded fashion, this physician has >20 years of experience assessing radiological outcomes for spinal arthrodesis. Future prospective studies utilizing randomization, blinding, more objective radiographic criteria, and clinical outcomes measures will present stronger support for the differences observed. Despite these study limitations, these data provide compelling evidence to suggest that a new hybrid osteoconductive bone graft, lacking calcium-based salts, may support rapid progression of bony fusion through endochondral ossification, even in patients with comorbidities to arthrodesis.

## Conclusion

Synthetic biomaterials utilized for spinal fusion procedures differ in composition and are often chosen by surgeons based upon handling characteristics. The present study compared two 510k cleared bone grafts utilized as extenders for posterolateral fusion in a series of 27 subjects where multiple radiographs obtained over the course of 15 months enabled side-by-side comparison of the radiographic appearance of each construct, with patients serving as their own control. Fusion success at the >12-month interval for sides receiving the radiolucent hybrid biomaterial, designed to support endochondral ossification, was statistically superior (*p* = 0.015) to contralateral sides receiving β-TCP. Given that each treatment side received similar quantities of autograft in the form of local bone, bone marrow aspirate and iliac crest, the observed treatment effect is surprising. Physicians must consider bone graft composition, biocompatibility, and more importantly mode of action in their decision to utilize specific biomaterial scaffolds. This concept becomes increasingly important when bone grafts are utilized as delivery vehicles for autologous cell therapies.

## Conflict of Interest Statement

Huston Davis Adkisson IV and Gary B. Gage are employees of ISTO Technologies, Inc. Geoffrey Stewart received financial support for statistical analysis and medical writing. Gary Neidert performed statistical analysis with financial support provided by ISTO Technologies, Inc. The reviewer, Konstantinos Markatos and handling Editor, Vassilios S. Nikolaou, declared their shared affiliation, and the handling Editor states that the process nevertheless met the standards of a fair and objective review.
